# The effect of emotional valence on concrete and abstract words in L2 lexical processing among Chinese–English learners

**DOI:** 10.3389/fpsyg.2026.1766572

**Published:** 2026-02-27

**Authors:** Ying Zhang, Zurui Zhang

**Affiliations:** 1 School of Foreign Studies, YanShan University, Qinhuangdao, Hebei, China; 2 Administration Office, Qinhuangdao Open University, Qinhuangdao, Hebei, China

**Keywords:** Chinese–English learners, concreteness, embodied cognition, emotional valence, L2 word processing

## Abstract

**Introduction:**

Emotional valence influences word processing, and this effect is modulated by word concreteness, but findings remain inconsistent in L2 contexts—especially among Chinese-English learners.

**Methods:**

Based on the Embodied Cognition Theory, we adopted a 3 (valence: positive/neutral/negative) × 2 (concreteness: abstract/concrete) mixed-factorial design and a lexical decision task to test 57 late Chinese-English bilinguals.

**Results:**

(1) The valence × concreteness interaction was significant in accuracy (emotion advantage was larger for concrete than abstract words) but not in reaction time; (2) The main effect of concreteness was significant: abstract words were processed faster and more accurately than concrete words.

**Discussion:**

Emotional valence exerts functionally different effects on L2 concrete/abstract word processing—concrete words benefit more from emotional facilitation via sensorimotor connections, while abstract words rely on emotional information to compensate for limited sensorimotor grounding.

## Introduction

Concreteness and emotional valence are important semantic variables affecting word recognition and processing. According to the dimension of concreteness, words can be divided into concrete words and abstract words. Concrete words (e.g., “hammer”) refer to specific, singular, and clearly bounded referents; abstract words (e.g., “freedom”) usually relate more to events, mental states, and contexts, and less to clearly definable and manipulable objects or entities (Barsalou et al., 2018). The Embodied Cognition Theory holds that the representation and processing of concrete words mainly rely on sensorimotor experiences in the physical world (Barsalou, 2008), while abstract words depend more on situational events and introspective information (Barsalou and Wiemer-Hastings, 2005). Emotional valence refers to the semantic dimension of words ranging from positive to negative, and is an important attribute of words. According to the dimension of valence, words can be classified into positive words, negative words, and neutral words. Studies have found that emotional words (positive and negative words) are generally processed faster and more accurately than neutral words (Kanske and Kotz, 2007; Kousta et al., 2009; Sheikh and Titone, 2016; Yao et al., 2018), and this processing advantage of emotional words is called the emotion effect (Kousta et al., 2011). In recent years, behavioral studies based on first language (L1) have found that the influence of emotional valence on word processing is modulated by the level of word concreteness. Specifically, when emotional valence and concreteness are considered simultaneously, they interactively affect word recognition and processing (Pauligk et al., 2019), and this interaction effect occurs in multiple cognitive tasks such as lexical decision, serial recall, and emotional priming (Barber et al., 2013; Kanske and Kotz, 2007; Pauligk et al., 2019; Sheikh and Titone, 2016; Yao et al., 2018). However, current research findings regarding the role of emotional valence in the processing of L1 concrete and abstract words remain inconclusive. Although these empirical studies have all confirmed the existence of an interaction between emotional valence and word concreteness, the patterns of this interaction vary. Therefore, the experimental conclusions related to word processing still require further verification and supplementation. Furthermore, empirical studies exploring the impact of emotional valence on the semantic processing of words with different concreteness from an L2 perspective are relatively scarce. Investigating this interaction in an L2 context is theoretically crucial because L2 processing is often characterized by ‘reduced emotional resonance’ (Pavlenko, 2012). Unlike L1, where emotional and sensorimotor grounding are established simultaneously during development, L2 words are often learned in academic settings with less sensory-emotional immersion. Determining whether the valence-concreteness interaction persists in L2 helps clarify whether these effects are universal features of the human semantic system or are dependent on the strength of original embodied experiences. Within the framework of the Embodied Cognition Theory, this study adopts a lexical decision task to examine the influence of emotional valence on the processing of concrete and abstract words by Chinese–English learners from the L1 and L2 perspectives, aiming to further clarify the role of emotion in the semantic processing of words with different concreteness and expand the understanding of the relationship between emotion and language.

For the processing of L1 words with different concreteness, early studies generally found the concreteness effect, that is, concrete words are usually processed faster and more accurately than abstract words. Moreover, the concreteness effect has been confirmed in a large number of empirical studies based on different experimental tasks (such as lexical decision, word naming, and sentence reading) and different languages (e.g., German, English, and Chinese) (Paivio, 1991; Schwanenflugel et al., 1992; Vigliocco et al., 2014). In recent years, the explanation of the concreteness effect has been challenged by the abstractness effect. After matching lexical attributes that play an important role in traditional theories, such as word imageability and context availability, Kousta et al. found that abstract words have a greater processing advantage than concrete words, that is, a reversal of the concreteness effect occurred. The researchers attributed this conclusion to the fact that abstract words have a stronger connection with emotional information and tend to be influenced by emotional valence (Kousta et al., 2011). Further evidence from neuroimaging shows that abstract words can more effectively activate brain regions related to emotional processing (Vigliocco et al., 2014). However, empirical studies that orthogonally manipulated emotional valence and concreteness found that emotional valence can also promote the processing of concrete words, and the studies also found that emotional valence and concreteness have an interactive effect in the semantic processing of words (Kanske and Kotz, 2007; Palazova et al., 2013; Yao et al., 2018). These findings cannot be explained by the stronger emotional basis of abstract words. On the contrary, they pointed out that emotional valence may have functional differences in the way it affects the processing of concrete and abstract words.

To date, regarding the role of emotional valence in the processing of L1 concrete and abstract words, there are two different viewpoints within the Embodied Cognition Theory. One viewpoint holds that the conceptual representation of emotion is essentially multi-modal. Perceiving an emotional stimulus (e.g., a smiling face, the word “smile”), simulating emotion-related bodily states (e.g., activating one’s own smiling muscles), and experiencing an emotion (e.g., feeling happy) all involve highly interconnected sensory, motor, and emotional systems (Niedenthal, 2007). Therefore, in the semantic processing of concrete words, the influence of emotional valence may be more pronounced than that on abstract words, because the former has a stronger connection with sensorimotor information. Among empirical studies investigating the interaction between emotional valence and concreteness, two Event-Related Potentials (ERP) studies based on lexical decision tasks support this viewpoint. Kanske and Kotz examined the concreteness effect and emotion effect of German nouns using a hemifield presentation technique through two lexical decision tasks, and recorded reaction times (RTs) and ERP data. The results found the emotion effect only in the processing of concrete words (Kanske and Kotz, 2007). Palazova et al. used a similar method to investigate the concreteness effect and emotion effect of German verbs with different valences in lexical decision tasks. The ERP results showed that there was an interactive effect between emotional valence and concreteness, and emotional valence only affected the processing of concrete words within 250–300 ms after stimulus onset (Palazova et al., 2013). Yao et al. examined the influence of emotional valence on the processing of English concrete and abstract words through a large-scale lexical decision task, and found that the emotion effect in concrete word processing was significantly greater than that in abstract word processing (Yao et al., 2018). The above studies provide behavioral and electrophysiological evidence for the viewpoint that emotional valence has a stronger impact on the processing of concrete words.

In contrast, another viewpoint represented by the embodiment views of semantic representation holds that abstract concepts cannot be embodied through sensory or motor information, and internal emotional experiences may be an alternative. It predicts a greater emotion effect in the semantic processing of abstract concepts (Vigliocco et al., 2009). This viewpoint highlights the connection between emotion and abstract concepts, and has been supported by numerous empirical studies from behavioral, electrophysiological, and brain neural research in recent years. Pauligk et al. investigated the interaction between emotional valence and concreteness in delayed lexical decision tasks through fMRI and electroencephalography (EEG) (Pauligk et al., 2019). Behavioral data showed that high emotional valence only promoted the accurate processing of abstract words; fMRI results revealed an interaction between concreteness and emotion in two central nodes of the semantic processing network: the left inferior frontal gyrus and the left middle temporal gyrus. The activation pattern of the left inferior frontal gyrus reflects a strong demand for semantic integration of abstract rather than concrete words, indicating that high emotional valence facilitates the selection of abstract words, and this effect is reversed in concrete words. Additionally, some studies have demonstrated the emotion effect only in abstract words. For example, an eye-tracking study by Sheikh and Titone found that higher emotional valence is more conducive to reading abstract words rather than concrete words (Sheikh and Titone, 2016). A lexical decision priming task by Yao and Wang found that only when Chinese abstract positive words were used as prime stimuli could they promote the recognition of target words with the same emotional valence (Yao and Wang, 2013). In the L2 domain, relevant evidence remains limited, but existing findings suggest that emotional effects may not fully mirror those observed in L1. Notably, Ferré et al. (2015) examined emotional valence and concreteness in bilingual word processing using paradigms closely related to lexical decision and reported patterns that have been interpreted as stronger emotional involvement for abstract words under certain conditions. This line of work highlights that bilingual populations, language-learning history, and task demands may shape the locus and direction of emotional modulation.

In summary, although scholars at home and abroad have conducted extensive research on the interaction between emotional valence and concreteness in word processing, there are still the following deficiencies. First, there are disagreements in academic circles regarding the conclusions on the role of emotional valence in the processing of L1 concrete and abstract words. Although all the above empirical studies have confirmed the existence of an interaction between emotional valence and concreteness, the patterns of this interaction are inconsistent. Therefore, the experimental conclusions on word processing still need further verification and improvement. Second, previous studies have mostly taken native speakers as research objects, lacking experimental evidence on the interactive influence of emotional valence and concreteness in L2 word processing. Moreover, there are no empirical studies in the existing literature involving the interactive influence of emotional valence and concreteness on L2 word processing among Chinese–English learners. Importantly, investigating the valence–concreteness interaction in L2 is not only a matter of filling an empirical gap. It provides a theoretically informative test bed for embodied and affective accounts developed largely from L1 research. For late L2 learners, lexical–semantic representations are typically less entrenched and may depend more on form-based access and learned mappings, while affective resonance and experiential grounding are often argued to be weaker or less automatic than in L1. Under this view, L2 processing constitutes a boundary condition that can reveal where and when emotional information enters lexical decisions: if the valence–concreteness interaction reflects universally available sensorimotor–affective simulations, it should remain robust in L2; if it relies on highly automatized affective grounding built through extensive lifetime experience in L1, the interaction may be attenuated, delayed, or expressed only in measures that capture later decision processes. Therefore, examining valence and concreteness in L2 can speak directly to the mechanisms underlying the interaction observed in L1, and can help determine whether emotional modulation is an early facilitation in lexical access or a later contribution to semantic evidence accumulation and decision certainty.

Therefore, it remains to be further verified whether the emotion effect in L2 word processing is modulated by word concreteness. Based on this, this study adopts a lexical decision task to examine the interaction between emotional valence and concreteness in L2 word processing among Chinese–English learners, respectively, from the perspectives of L2, aiming to answer the following questions: Is the emotion effect in L2 word processing of Chinese–English learners influenced by word concreteness? If so, what is the influence? Based on existing studies, this study hypothesizes: (1) Due to their stronger connection with sensorimotor information, there will be a stronger emotion effect in the processing of concrete words; (2) Conversely, due to their stronger connection with emotional information, the emotion effect will be amplified in the processing of abstract words.

## Method

### Participants

The sample size was determined via *a priori* power analysis using G*Power 3.1 (Faul et al., 2007). For a two-way repeated-measures ANOVA (2 × 3 design) with *α* = 0.05, power (1 − *β*) = 0.80, we estimated a medium effect size of *f* = 0.25 based on Yao et al. (2018), a seminal study with the identical experimental design (valence × concreteness) and lexical decision task paradigm to the present study. Specifically, Yao et al. (2018) reported the regression coefficient (*b* ≈ −1.7) and standard error (SE ≈ 0.6) for the valence × concreteness interaction effect (reaction time index) in their linear mixed-effects model (LMM) results. We derived the effect size through three standardized statistical steps: (1) Calculated the *t*-value of the interaction effect: *t* ≈ 2.83; (2) Converted the *t*-value to partial eta-squared using the formula for within-subjects designs: *ηp*^2^ = *t*^2^/(*t*^2^ + df), df ≈ 126 (approximated as sample size − 1), yielding *ηp*^2^ ≈ 0.06; (3) Transformed *ηp*^2^ to Cohen’s *f* via the universal conversion formula (Cohen, 2013): *f* = $\sqrt{\frac{\mathrm{\eta p}2}{\left(1-\mathrm{\eta p}2\right)}}$ ≈ 0.25. This value is consistent with Cohen’s classic definition of a medium effect size. Based on this parameter setting, the required minimum sample size was 48. This experiment recruited 57 non-English major undergraduate students from a university in China as participants. The average age of the participants (27 males and 30 females) was 19.14 years (SD = 0.55), and all scored above 33 points (*M* = 43.33) on the Quick Placement Test (QPT). All participants were right-handed, with normal or corrected-to-normal vision, and no history of brain injury or neurological disorders. According to the questionnaire results, the participants began learning Chinese at birth, and their average English learning duration was 11.09 years (SD = 0.97). Some researchers argued that individuals who acquire a second language after the age of six are late bilinguals (Yao and Wang, 2013), so the participants in this study were late Chinese–English bilinguals. All participants volunteered to participate in the experiment and received a certain amount of remuneration upon completion.

### Materials

This experiment included a total of 120 words, and the target words were selected from 270 words in the appendix of Yao et al.’s study (Yao et al., 2018). Data on concreteness, valence, and arousal were all derived from this source. The study adopted a 3 (valence: positive, neutral, negative) × 2 (word concreteness: abstract, concrete) mixed-factorial experimental design, resulting in 6 experimental conditions with 20 words under each condition. Half of the 120 words were relatively concrete in meaning (e.g., “toy”), and the other half were relatively abstract (e.g., “trust”). Under each concreteness condition, one-third were positive words (e.g., “elegance,” “smile”), one-third were neutral words (e.g., “method,” “road”), and one-third were negative words (e.g., “anger,” “prison”). This study controlled for variables such as word length, word frequency, valence, arousal, imageability, and familiarity. The word length of the 6 categories of words was identical, and their word frequencies^1^ (sourced from the BNC corpus) were highly similar. Concrete and abstract words were highly matched in terms of valence and arousal. In terms of valence, there were significant differences between each pair of positive, negative, and neutral words (*ps* < 0.001), while no significant differences were found between abstract positive words and concrete positive words, or between abstract negative words and concrete negative words (*ps* > 0.05). In terms of arousal, positive and negative words each showed significant differences from neutral words (*ps* < 0.001), while no significant differences were observed among the 4 categories of emotional words (*ps* > 0.05). Twenty non-participants (with a proficiency level comparable to that of the formal participants) evaluated the familiarity of 270 initially selected words from Yao et al. (2018) using a 5-point Likert scale (1 = completely unfamiliar to 5 = extremely familiar). We excluded words with familiarity ratings below 4.5 to ensure high familiarity (a critical control for L2 word processing). The remaining 120 words were evenly divided into 6 experimental conditions (20 words per condition), and a one-way ANOVA confirmed no significant differences in familiarity across conditions (*F*(5, 114) = 0.82, *p* = 0.54). In addition, 120 pronounceable and orthographically legal pseudowords were created as distractors by replacing one or two letters of each of the 120 real words (e.g., femper, temice), and the pseudowords matched the target words in string length. The properties of the 6 categories of target words are shown in Table 1.

**Table 1:** List of relevant attributes for target words (L2: English).

Lexical Attributes	Abstract word			Concrete word		
	Positive	Neutral	Negative	Positive	Neutral	Negative
Number	20	20	20	20	20	20
Valence	7.40 (0.52)	5.26 (0.25)	2.28 (0.76)	7.16 (0.56)	5.22 (0.36)	2.58 (0.83)
Concreteness	2.81 (0.43)	2.98 (0.49)	2.87 (0.36)	6.07 (0.50)	6.14 (0.43)	5.97 (0.60)
Frequency	34.48 (33.90)	37.67 (33.54)	37.83 (47.50)	34.19 (38.58)	37.75 (44.69)	34.25 (34.45)
Length	5.80 (1.47)	5.80 (1.47)	5.80 (1.47)	5.80 (1.58)	5.80 (1.58)	5.80 (1.58)
Arousal	5.65 (0.81)	3.79 (0.54)	5.56 (0.60)	5.91 (0.68)	3.97 (0.56)	5.51 (0.95)
Imageability	3.44 (0.73)	2.82 (0.45)	3.32 (0.64)	6.39 (0.45)	6.33 (0.25)	6.09 (0.52)
Familiarity	4.74 (0.36)	4.73 (0.32)	4.70 (0.41)	4.88 (0.28)	4.74 (0.30)	4.84 (0.35)

The measurement units are as follows: Affective valence from 1 (very negative) to 5 (neutral) to 9 (very positive); Specificity from 1 (very abstract) to 7 (very concrete); Word frequency: occurrence rate per million; Word length: number of letters; Arousal level from 1 (very weak) to 5 (neutral) to 9 (very strong); Imagery from 1 (very difficult to imagine) to 7 (very easy to imagine); and Familiarity from 1 (very unfamiliar) to 5 (very familiar). Data in parentheses indicate standard deviation.

### Procedures

**Figure 1:**
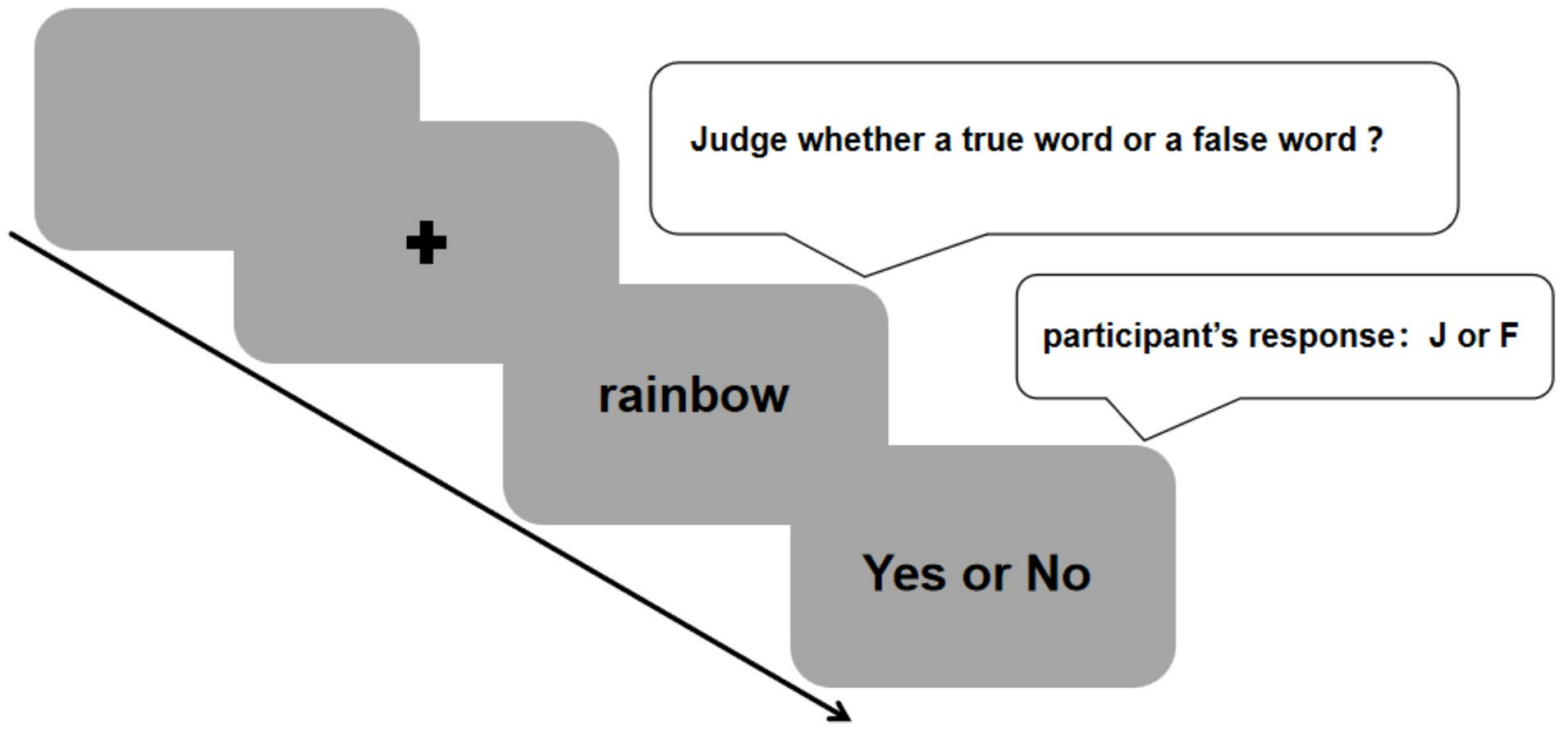
The procedure of an example trial of Experiment. Each trial in the experiment started with a blank screen lasting 500 ms; then a black fixation cross appeared at the center of the screen for 1,500 ms; subsequently, the black fixation cross was replaced by the target word, and participants needed to make a rapid judgment on the type of the word. If judging the word as a real word, they pressed Key J on the keyboard; if judging it as a pseudoword, they pressed Key F, and then proceeded to the next trial.

This experiment adopted the lexical decision task paradigm, with experimental materials including the aforementioned 120 English real words and 120 English pseudowords. The study used Psychopy.2025.2.0 (Peirce, 2007) software to program the experimental procedure. Target words appeared on the screen without any inflectional changes, using Times New Roman, black, 24-point font. The 240 experimental words were divided into two blocks for the experiment in pseudorandom order (120 words per block), with each block containing an equal number of real words and pseudowords, and words under the same condition did not appear consecutively more than three times. Before the formal experiment, participants were informed that the target words included both real words and pseudowords, and their task was to press the corresponding keys on the keyboard as quickly and accurately as possible. Each trial in the experiment started with a blank screen lasting 500 ms; then a green fixation cross appeared at the center of the screen for 1,500 ms; subsequently, the green fixation cross was replaced by the experimental target word, and participants needed to make a rapid judgment on the type of the word. If judging the word as a real word, they pressed Key ‘J’ on the keyboard; if judging it as a pseudoword, they pressed Key ‘F’, and then proceeded to the next trial. If the participant did not respond within 2,000 ms after the target word was presented, the system would automatically move to the next trial. Participants familiarized themselves with the experimental procedure through a practice block before the formal experiment, and the words in the practice block did not appear in the formal experiment. The experiment was divided into three blocks, and participants could take a break between two blocks. During the practice phase, immediate feedback was provided for participants’ key presses, but no feedback was given during the formal experiment. The total experimental time for each participant was approximately 25 min. The experimental procedure of the present study was shown in Figure 1.

### Transparency and openness statement

The materials and datafiles of this article will be publicly shared upon publication. The complete dataset will be available via the Open Science Framework.^2^ None of the experiments was preregistered.

## Results

All participants achieved an accuracy rate of over 80%. For the analysis of reaction time data, the following data were excluded: incorrect trials and unanswered trials were deleted first, and then trials with reaction times less than 250 ms or greater than 1,500 ms were excluded. The 250-ms cutoff is consistent with standard practices in L2 lexical decision tasks (Yao et al., 2018), as reactions faster than this threshold typically do not reflect intentional lexical processing (i.e., participants cannot visually encode and semantically evaluate a word within 250 ms). Finally, for each participant under each condition, trials with reaction times exceeding two standard deviations were excluded. The removal of incorrect trials and outliers resulted in an average loss of approximately 17% of reaction time data per participant.

**Figure 2:**
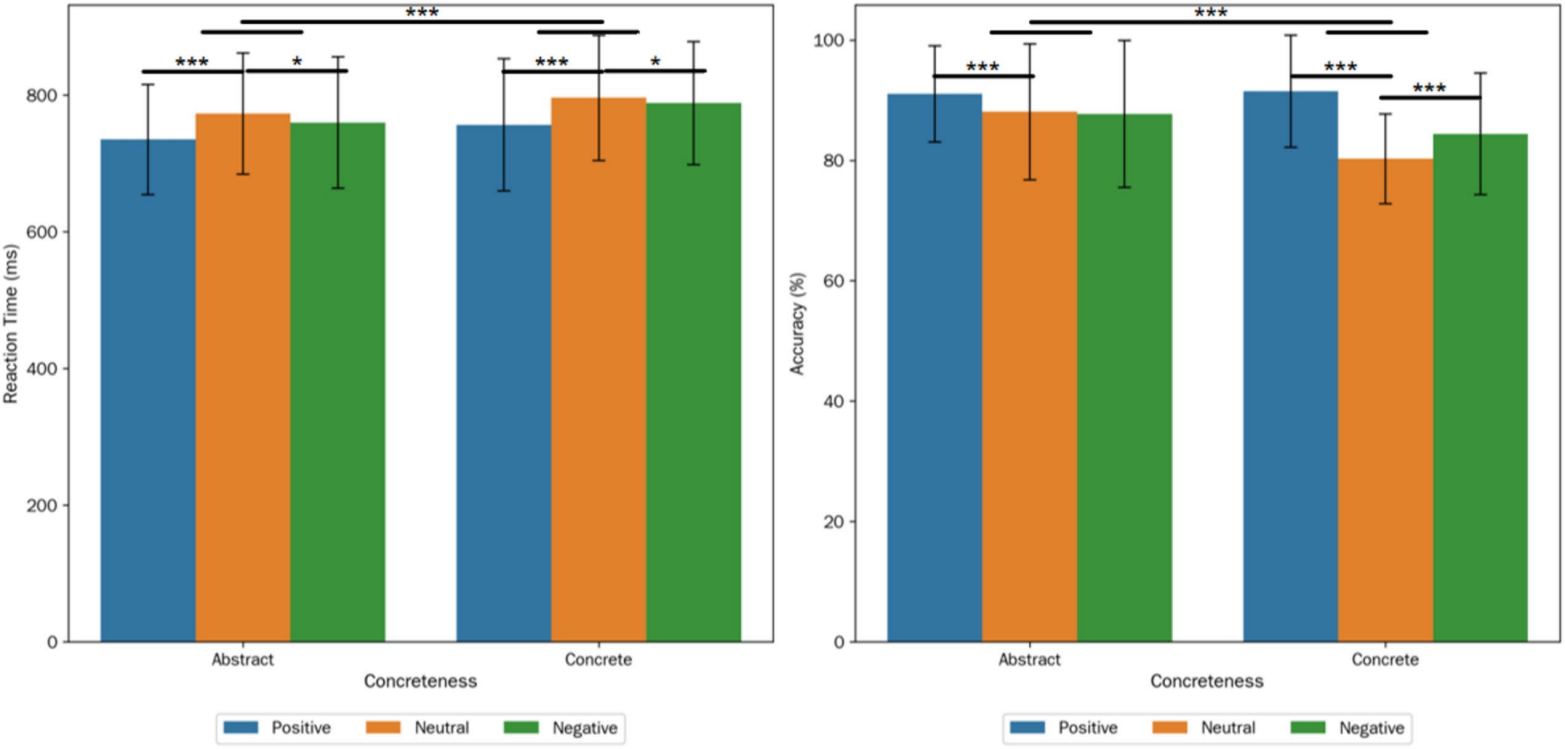
The reaction time (RT) and the accuracy rate (ACC) of six types of lexical processing when subjects were treated as random variables in our experiment. Error bars represent standard errors of the mean across participants. * represents *p* < 0.05, *** represents *p* < 0.001.

SPSS 26.0 statistical software (Kusumah, 2018) was used to conduct two-way repeated-measures analysis of variance (ANOVA) on the valid data, with 2 (concreteness: abstract, concrete) × 3 (valence: positive, neutral, negative) as the factors and participants as the random variable. Both word concreteness and valence were within-subject variables, and reaction time was the dependent variable (Greenhouse–Geisser correction was applied when the sphericity assumption was violated). The results showed: (1) The main effect of valence was extremely significant (*F*(2, 112) = 34.990, *p* < 0.001, *ηp*^2^ = 0.385). When participants were taken as the random variable, the reaction speed of participants to neutral words was significantly slower than that to negative words (*p* = 0.046) and positive words (*p* < 0.001). Among emotional words, the reaction time of positive word processing was significantly shorter than that of negative words (*p* < 0.001); (2) The main effect of word concreteness was significant (*F*(1, 56) = 11.043, *p* < 0.001, *ηp*^2^ = 0.165). Among them, the reaction time of abstract word processing was significantly shorter than that of concrete words; (3) The two-way interaction between word concreteness and valence was not significant (*F*(2, 112) = 0.272, *p* = 0.760, *ηp*^2^ = 0.005). These results were shown in Figure 2. For reaction time, the result pattern of by-items analysis (materials were taken as the random variable) was completely consistent with that of by-subjects analysis (participants were taken as the random variable).

Similarly, SPSS 26.0 was used to conduct a two-way repeated-measures analysis of variance (ANOVA) on the accuracy rates of the available participant data, with 2 (concreteness: abstract, concrete) × 3 (valence: positive, neutral, negative) as the factors. Both word concreteness and valence were within-subject variables, and accuracy rate was the dependent variable. The results with participants as the random variable showed: (1) The main effect of valence was significant (*F*(2, 112) = 31.149, *p* < 0.001, *ηp*^2^ = 0.357). The accuracy rate of positive word processing was significantly higher than that of neutral words (*p* < 0.001); the accuracy rate of negative word processing was marginally significantly higher than that of neutral words (*p* = 0.049); among emotional words, the accuracy rate of positive word processing was significantly higher than that of negative words (*p* < 0.001); (2) The main effect of word concreteness was extremely significant (*F*(1, 56) = 19.068, *p* < 0.001, *ηp*^2^ = 0.254), and the accuracy rate of abstract word processing was significantly higher than that of concrete words; (3) There was a significant interaction effect between word concreteness and valence (*F*(2, 112) = 10.622, *p* < 0.001, *ηp*^2^ = 0.159). Further simple effect analysis revealed: (1) In abstract word processing, the accuracy rate of positive words was significantly higher than that of neutral words (*p* < 0.001); the accuracy rate of positive words was significantly higher than that of negative words (*p* < 0.001); there was no significant difference between neutral words and negative words (*p* = 0.787). In concrete word processing, the accuracy rates of both positive and negative words were significantly higher than that of neutral words (*ps* < 0.001), and the accuracy rate of positive words was significantly higher than that of negative words (*p* < 0.001). It can be seen that compared with abstract words, emotional words (positive and negative words) had a greater processing advantage over neutral words in concrete word processing, and the emotion effect was more significant; (2) Under the positive valence condition, there was no significant difference in the processing accuracy rates between abstract and concrete words (*p* = 0.593); under the neutral and negative valence conditions, the processing accuracy rates of abstract words were significantly higher than those of concrete words (*ps* < 0.05). These results were shown in Figure 2. For accuracy, the by-items analysis (materials were taken as the random variable) showed a significant main effect of valence, *F*(2, 114) = 3.326, *p* = 0.046, *ηp*^2^ = 0.149, which was consistent with the result of by-subjects analysis. The main effect of concreteness (*F*(1, 114) = 2.45, *p* = 0.121, *ηp*^2^ = 0.041) and the interaction between valence and concreteness (*F*(2, 114) = 1.18, *p* = 0.303, *ηp*^2^ = 0.020) were not significant. Post-hoc comparisons (Bonferroni-corrected) of the valence main effect in the by-items analysis revealed that the accuracy of positive word processing was significantly higher than that of neutral words (*p* < 0.001); the accuracy of negative word processing was significantly higher than that of neutral words (*p* < 0.001); and the accuracy of positive word processing was significantly higher than that of negative words (*p* < 0.001).

## Discussion

The present study, grounded in the framework of Embodied Cognition Theory, aimed to explore the interactive effects of emotional valence and word concreteness on the processing of second language (L2, English) concrete and abstract words among Chinese–English learners through the lexical decision task experiment. The findings revealed distinct patterns of interaction between the two variables across the L2 contexts. Specifically, our results demonstrated that the interaction between emotional valence and concreteness was reflected in accuracy: emotional concrete words (both positive and negative) exhibited a more prominent processing advantage over neutral concrete words, and this emotional effect was stronger than that in abstract words. Additionally, L2 processing showed an abstractness effect, with abstract words processed faster than concrete words. Below, we will analyze and interpret these findings in detail from the perspectives of L2 word processing, integrating relevant theoretical frameworks and existing research evidence. The following discussion centered on these findings.

### The effect of lexical specificity on emotional effects of second language (L2) lexical processing

The present study showed that the interaction between emotional valence and concreteness was reflected in accuracy. The emotion effect in L2 concrete word processing was significantly greater than that in abstract word processing, which verified the first research hypothesis proposed earlier. Our experiment found that the main effect of word concreteness was significant in terms of reaction time, and abstract words were processed faster than concrete words. The results of the accuracy analysis with participants as the random variable showed a significant interaction between emotional valence and concreteness. Compared with abstract words, emotional valence exerted a greater facilitating effect on the accurate processing of L2 concrete words. This is consistent with the reaction time and accuracy results of Yao et al. (2018). Their reaction time analysis based on the lexical decision task revealed a significant interaction between word concreteness and valence, with the emotion effect in concrete word processing being significantly greater than that in abstract word processing. However, it is inconsistent with the research results of Pauligk et al. (2019). Through functional magnetic resonance imaging (fMRI) and electroencephalography (EEG), Pauligk et al. (2019) investigated the interaction between emotional valence and concreteness in delayed lexical decision tasks. Their behavioral data showed that high positive and negative valence promoted the accurate processing of abstract words but had no facilitating effect on the processing of concrete words. The stronger emotion effect in concrete word processing may stem from their higher concreteness and imageability, i.e., richer connections with sensorimotor experiences. Although concreteness and imageability are highly correlated, they at least partially reflect different aspects of word semantics. Concreteness involves the categorical ontological distinction between material entities and conceptual entities, and it is bimodally distributed; in contrast, imageability is unimodally distributed, reflecting the degree of association between words and sensory (mainly visual) motor information (Yao et al., 2018). Words with high imageability are semantically richer and generate more semantic activation during semantic processing (i.e., richer sensory simulation and richer motor simulation). The Embodied Cognition Theory holds that conceptual processing and perceptual-motor processes share a common neural basis. The partial activation of processing circuits in the conceptual system (e.g., seeing a venomous snake or reading the word “venomous snake”) may cascade to the complementary activation of other parts of the circuit (e.g., feeling fear). In other words, emotional activation can benefit from the activation of sensorimotor information, so the high emotional experience information of concrete emotional words can better activate participants’ sensory and perceptual systems. Further electrophysiological evidence indicated that concrete emotional words and neutral words activated mental imagery differently. The processing of concrete neutral words activated semantic and sensorimotor systems, while the processing of concrete emotional words simultaneously activated lexical-semantic and emotional systems (Kanske and Kotz, 2007). This dual effect of emotion and perception prompts participants to respond more accurately to emotional words. In addition, when participants acquire concrete emotional words, the emotional responses triggered by these words are combined with specific perceptual experiences, forming deeper memory traces. Subsequently, when processing concrete words with emotional valence (e.g., “smile”), they may associate them with scenes of themselves or others smiling. This interaction between specific perceptual experiences and positive emotional responses enhances the word recognition effect.

In addition, our findings should be discussed in relation to Ferré et al. (2015), who examined valence and concreteness in an L2/bilingual context using a novel word learning paradigm. In their study, proficient Catalan–Spanish bilinguals first learned Basque words paired with Spanish translations, and were subsequently tested using a Basque go/no-go lexical decision task and a backward translation task, both immediately after learning and one week later. Ferré et al. (2015) reported an interaction suggesting that emotionality particularly benefited abstract words (with a reliable advantage for negative abstract items) in this early learning context. Importantly, the goals and processing demands in Ferré et al. (2015) differ from those of the present study: whereas Ferré et al. (2015) targeted the initial establishment and consolidation of new L2 lexical–semantic links (where emotion may scaffold abstract concepts that lack strong sensorimotor grounding), our study examined established L2 lexical access under speeded lexical decision demands. This difference in learning stage and task requirements may help explain why emotional modulation is observed as an abstract-word advantage in some learning-based bilingual paradigms, but emerges as enhanced decision accuracy for concrete words in our late L2 lexical decision data.

In the current study, the central result was that emotional valence interacted with concreteness in accuracy but not in reaction time. Specifically, the valence × concreteness interaction was not significant for RT, whereas it was robust for accuracy, with emotional facilitation being larger for concrete than for abstract words and the lowest accuracy observed for neutral concrete words. This dissociation is theoretically meaningful in an L2 context because RT and accuracy may tap partially different processing components under speeded lexical decision demands. Under time pressure, RT is often dominated by early-stage processes such as orthographic identification and initial lexical access. For late L2 learners, these early stages may rely more heavily on form-based cues and learned lexical mappings, whereas affective resonance may be less automatic and therefore less likely to yield measurable facilitation in response speed. In contrast, accuracy can reflect the quality of accumulated semantic evidence and the certainty of the word/nonword decision. Emotional valence may enhance this evidence accumulation—particularly for concrete words whose meanings are more tightly linked to sensorimotor and image-based representations—thereby improving correct responding without necessarily accelerating the initial access stage. This account also explains why emotional facilitation was most visible for concrete items: when concreteness provides richer perceptual–experiential structure, emotional information can add discriminative support to the lexical decision, reducing confusability with pseudowords and strengthening the decision signal. Neutral concrete words, lacking affective support while still engaging rich semantic content, may be especially susceptible to uncertainty under speeded decisions, consistent with their comparatively lower accuracy in our data. Overall, our findings suggest that, in late L2 processing, emotional valence may contribute more reliably to later decision-related stages than to early access speed, producing an interaction that emerges in accuracy but not in RT. Additionally, no more significant emotion effect was found in the reaction time of concrete word processing, which might be related to the experimental paradigm. Studies have shown that when words are presented out of context and under time pressure (as in this experiment), emotional valence may lead to additional processing load in the semantic selection process. This situation may occur if lexical attributes other than emotional valence provide sufficient cues for the retrieval of lexical semantic information (Pauligk et al., 2019). In the processing of concrete words, there are sufficient lexical semantic retrieval cues (such as imageability and concreteness) in the early processing stage. Therefore, the subsequent semantic retrieval triggered by emotional valence may result in an excess of semantic information, thereby increasing the processing cost of concrete emotional words and causing the disappearance of the processing advantage of concrete emotional words (Pauligk et al., 2019).

The results of the current study showed that after strictly matching variables such as valence and arousal of concrete and abstract words, abstract words were processed faster than concrete words, exhibiting an abstractness effect in reaction time. This was inconsistent with the research results of the previous studies (Kanske and Kotz, 2007; Pauligk et al., 2019; Yao et al., 2018). They all found the concreteness effect in studies investigating the interaction between emotional valence and concreteness with L1 as the research object. This indicates that the way emotional valence influences the processing of L2 concrete and abstract words may differ from that of L1, and the underlying processing mechanism remains to be further explored in the future. However, the finding of the abstractness effect in L2 word processing supports the Affective Embodiment Account (AEA) (Kousta et al., 2011; see also Borghi et al., 2017, for a review). The AEA hypothesizes that abstract words carry more emotional information than concrete words, and emotion (non-linguistic) may provide a guiding mechanism for the representation and processing of abstract words to compensate for the lack of direct mapping of sensory experiences. After matching other attributes of words, the high emotional experience information of abstract words makes their emotional connections with abstract target words closer in the semantic network, so it is easier to activate similar emotional nodes, thereby promoting the recognition of abstract target words (Yao and Wang, 2013). The hypothesis that abstract words carry more emotional information can be confirmed in another aspect: people are more inclined to use abstract words to express their feelings and experiences. For example, people use the abstract word “freedom” to express their positive, excited, open and other emotions. This research result is also consistent with the relevant hypotheses of the Embodied Cognition Theory on the representation and processing of abstract words, that is, the representation and processing of abstract words rely more on situational events and introspective information, especially emotion.

Moreover, our results align with the view that emotional facilitation can be more pronounced for concrete words, at least in the accuracy dimension under speeded L2 lexical decision. At the same time, the broader literature includes evidence supporting stronger emotional involvement for abstract words under other conditions (e.g., Kousta et al., 2011). Rather than implying a single uniform direction for the valence–concreteness interaction, our findings suggest that the relative emotional advantage for abstract versus concrete words may be conditional on (1) language experience and the strength of affective grounding (L1 vs. late-learned L2), and (2) task demands that determine whether emotional information can influence early access speed or later semantic/decision stages. From this perspective, the ‘abstract-word emotion effect’ may generalize more readily to contexts that encourage deeper conceptual integration or in languages/populations where affective resonance is highly automatized, whereas in late L2 lexical decision, emotional modulation may surface more strongly as improved decision accuracy for concrete words.

Overall, our experiment of this study examined the effects of emotional valence and concreteness on L2 word processing among Chinese–English learners in the lexical decision task. The results showed that an emotion effect emerged in L2 word processing, and this emotion effect was modulated by word concreteness. Specifically, emotional valence exerted a greater facilitating effect on the accurate processing of L2 concrete words, which may be attributed to the stronger association between concrete words and sensorimotor information. Our experiment also found that when variables such as valence and arousal of concrete and abstract words were item-wise matched, participants recognized abstract words faster than concrete words, producing an abstractness effect. This result supported the AEA, namely that the high emotional experience information of abstract words could exert a significant facilitating effect in the process of word recognition. This result of our experiment was not entirely consistent with the conclusions of any previous study, but it was not contradictory to the relevant views of the Embodied Cognition Theory on lexical semantic processing—specifically, the representation and processing of concrete words mainly relied on sensorimotor experiences in the physical world, while abstract words depended more on situational events and introspective information, especially emotion.

## Limitation and future direction

Due to the limitations of article length and research conditions, this study did not examine the impact of differences in participants’ L2 proficiency on the experimental results; in addition, it did not consider age of acquisition, a factor known to affect visual word processing. Future research can further investigate the effects of these two factors on the emotion effect in L2 word processing. Also, this study mainly explored the impact of emotional valence on the processing of concrete and abstract words in L2 through behavioral data such as reaction time and error rate, but failed to investigate differences in their processing mechanisms through electrophysiological and cognitive neuroimaging evidence. Future research can combine technical methods such as Event-Related Potentials (ERPs) and functional Magnetic Resonance Imaging (fMRI) to further explore these questions.

In addition, the core finding of the significant valence × concreteness interaction in accuracy was only observed in the by-subjects analysis (participants as the random variable), but not in the by-items analysis (materials as the random variable). This indicates that the interaction effect may lack sufficient robustness at the item level, which could be attributed to two main reasons: (1) Although we strictly matched lexical attributes (e.g., word frequency, length, familiarity) across six experimental conditions, there may still be subtle individual differences in the emotional salience and concreteness of individual target words, which may weaken the interaction effect at the item level; (2) The number of target words per condition (20 words) may be relatively limited for the by-items analysis, and a larger sample of experimental items may help to verify the robustness of the valence × concreteness interaction effect. Future research can expand the number of experimental items and further screen lexical materials to reduce individual item differences, so as to improve the consistency of by-subjects and by-items analysis results. In addition, the non-significant interaction in by-items analysis does not negate the core conclusion of this study, but it reminds us that the valence-concreteness interaction in L2 lexical processing is affected by both participant factors and item factors, and the item-level modulation needs to be further explored.

## Theoretical and practical implications

This study advances the Embodied Cognition Theory by verifying that the valence-concreteness interaction persists in late L2 processing (Chinese–English learners) with a unique pattern (significant in accuracy but not reaction time), which also reconciles divergent findings in existing L2 research (e.g., Ferré et al., 2015) by clarifying the moderating role of learning stage and task demands. Meanwhile, the observed abstractness effect provides additional empirical support for the AEA in the L2 context, enriching the bilingual lexical processing literature with new evidence. For L2 English vocabulary teaching of Chinese–non-English major undergraduates, these findings offer targeted embodied teaching strategies: for concrete words, integrate emotional situational simulation and sensorimotor experience to strengthen their affective-sensorimotor links and improve processing accuracy; for abstract words, emphasize their emotional connotations and introspective associations to leverage the abstractness effect in processing speed. In addition, prioritizing high-familiarity vocabulary in teaching and incorporating simplified lexical decision tasks into practice can reduce learners’ cognitive load and maximize the emotional modulation effect on L2 lexical processing, providing actionable insights for college English vocabulary teaching that aligns with the cognitive characteristics of late Chinese–English learners.

## Conclusion

Grounded in Embodied Cognition Theory, this study explored the interaction of emotional valence and word concreteness in second language (L2, English) lexical processing among Chinese–English learners via a lexical decision task, and uncovered distinct cross-linguistic patterns in the emotional modulation of lexical processing. In L2 processing of this study, the interaction between emotional valence and concreteness was observed in accuracy. Concrete emotional words—both positive and negative—exhibited a more pronounced processing advantage over neutral words relative to abstract emotional words. An abstractness effect also emerged, with abstract words recognized faster than concrete words following the matching of valence and arousal. This result supported the AEA, which emphasizes that the emotional experiential information of abstract words compensates for their limited sensorimotor mappings. These results confirmed functional differences in the influence of emotional valence across L2 lexical processing. In L2, it yielded a stronger emotional effect for concrete words—linked to their robust sensorimotor connections—and promoted abstract word processing through emotional information. Consistent with Embodied Cognition Theory, concrete words were grounded in sensorimotor experiences for their representation and processing, whereas abstract words were predominantly underpinned by situational and introspective information, particularly emotion. Unexamined individual differences, including L2 proficiency and age of acquisition, constituted a limitation of the study. This study enriched bilingual literature by uncovering cross-linguistic differences in the emotional modulation of lexical processing and provided empirical support for embodied cognition in bilingual contexts.

## Data Availability

The datasets presented in this study can be found in online repositories. The names of the repository/repositories and accession number(s) can be found at: https://osf.io/4ewj2.

## Footnotes

1
http://www.natcorp.ox.ac.uk

2
https://osf.io/4ewj2

## References

[ref1] Barber H. A. Otten L. J. Kousta S.-T. Vigliocco G. (2013). Concreteness in word processing: ERP and behavioral effects in a lexical decision task. Brain Lang. 125, 47–53. doi: 10.1016/j.bandl.2013.01.005, 23454073

[ref2] Barsalou L. W. (2008). Grounded cognition. Annu. Rev. Psychol. 59, 617–645. doi: 10.1146/annurev.psych.59.103006.093639, 17705682

[ref3] Barsalou L. W. Dutriaux L. Scheepers C. (2018). Moving beyond the distinction between concrete and abstract concepts. Philos. Trans. R. Soc. B. 373:20170144. doi: 10.1098/rstb.2017.0144, 29915012 PMC6015837

[ref4] Barsalou L. W. Wiemer-Hastings K. (2005). “Situating abstract concepts” in Grounding cognition. eds. Pecher D. Zwaan R. A.. 1st ed. (New York, USA: Cambridge University Press), 129–163.

[ref5] Borghi A. M. Binkofski F. Castelfranchi C. Cimatti F. Scorolli C. Tummolini L. (2017). The challenge of abstract concepts. Psychol. Bull. 143, 263–292. doi: 10.1037/bul0000089, 28095000

[ref6] Cohen J. (2013). Statistical power analysis for the behavioral sciences. Hillsdale, New Jersey, USA: Routledge.

[ref7] Faul F. Erdfelder E. Lang A. G. Buchner A. (2007). G*power 3: a flexible statistical power analysis program for the social, behavioral, and biomedical sciences. Behav. Res. Methods 39, 175–191. doi: 10.3758/BF03193146, 17695343

[ref8] Ferré P. Ventura D. Comesaña M. Fraga I. (2015). The role of emotionality in the acquisition of new concrete and abstract words. Front. Psychol. 6:976. doi: 10.3389/fpsyg.2015.00976, 26217289 PMC4497307

[ref9] Kanske P. Kotz S. A. (2007). Concreteness in emotional words: ERP evidence from a hemifield study. Brain Res. 1148, 138–148. doi: 10.1016/j.brainres.2007.02.044, 17391654

[ref10] Kousta S.-T. Vigliocco G. Vinson D. P. Andrews M. Del Campo E. (2011). The representation of abstract words: why emotion matters. J. Exp. Psychol. Gen. 140, 14–34. doi: 10.1037/a0021446, 21171803

[ref11] Kousta S.-T. Vinson D. P. Vigliocco G. (2009). Emotion words, regardless of polarity, have a processing advantage over neutral words. Cognition 112, 473–481. doi: 10.1016/j.cognition.2009.06.007, 19591976

[ref12] Kusumah E. P. (2018). Technology acceptance model (TAM) of statistical package for the social sciences (SPSS) applications. Integr. J. Bus. Econ. 2:1. doi: 10.33019/ijbe.v2i1.47

[ref13] Niedenthal P. M. (2007). Embodying emotion. Science 316, 1002–1005. doi: 10.1126/science.1136930, 17510358

[ref14] Paivio A. (1991). Dual coding theory: retrospect and current status. Can. J. Psychol./Revue Canadienne de Psychologie 45, 255–287. doi: 10.1037/h0084295

[ref15] Palazova M. Sommer W. Schacht A. (2013). Interplay of emotional valence and concreteness in word processing: an event-related potential study with verbs. Brain Lang. 125, 264–271. doi: 10.1016/j.bandl.2013.02.008, 23578815

[ref16] Pauligk S. Kotz S. A. Kanske P. (2019). Differential impact of emotion on semantic processing of abstract and concrete words: ERP and fMRI evidence. Sci. Rep. 9:14439. doi: 10.1038/s41598-019-50755-3, 31594966 PMC6783415

[ref17] Pavlenko A. (2012). Affective processing in bilingual speakers: disembodied cognition? Int. J. Psychol. 47, 405–428. doi: 10.1080/00207594.2012.743665, 23163422

[ref18] Peirce J. W. (2007). PsychoPy—psychophysics software in Python. J. Neurosci. Methods 162, 8–13. doi: 10.1016/j.jneumeth.2006.11.017, 17254636 PMC2018741

[ref19] Schwanenflugel P. J. Akin C. Luh W.-M. (1992). Context availability and the recall of abstract and concrete words. Mem. Cogn. 20, 96–104. doi: 10.3758/BF03208259, 1549068

[ref20] Sheikh N. A. Titone D. (2016). The embodiment of emotional words in a second language: an eye-movement study. Cognit. Emot. 30, 488–500. doi: 10.1080/02699931.2015.1018144, 25786993

[ref21] Vigliocco G. Kousta S.-T. Della Rosa P. A. Vinson D. P. Tettamanti M. Devlin J. T. . (2014). The neural representation of abstract words: the role of emotion. Cereb. Cortex 24, 1767–1777. doi: 10.1093/cercor/bht025, 23408565

[ref22] Vigliocco G. Meteyard L. Andrews M. Kousta S. (2009). Toward a theory of semantic representation. Lang. Cogn. 1, 219–247. doi: 10.1515/LANGCOG.2009.011

[ref23] Yao B. Keitel A. Bruce G. Scott G. G. O’Donnell P. J. Sereno S. C. (2018). Differential emotional processing in concrete and abstract words. J. Exp. Psychol. Learn. Mem. Cogn. 44, 1064–1074. doi: 10.1037/xlm0000464, 29431458

[ref24] Yao Z. Wang Z. (2013). The effects of the concreteness of differently valenced words on affective priming. Acta Psychol. 143, 269–276. doi: 10.1016/j.actpsy.2013.04.008, 23684852

